# Disruption of *CYP88B1* by transcription activator-like effector nuclease in potato and potential use to produce useful saponins

**DOI:** 10.5511/plantbiotechnology.24.0614a

**Published:** 2024-09-25

**Authors:** Shuhei Yasumoto, Hyoung Jae Lee, Ryota Akiyama, Satoru Sawai, Masaharu Mizutani, Naoyuki Umemoto, Kazuki Saito, Toshiya Muranaka

**Affiliations:** 1 Department of Biotechnology, Graduate School of Engineering, Osaka University, Yamadaoka, Suita, Osaka 565-0871, Japan; 2 Industrial Biotechnology Initiative Division, Institute for Open and Transdisciplinary Research Initiatives, Osaka University, Yamadaoka, Suita, Osaka 565-0871, Japan; 3 Graduate School of Agricultural Science, Kobe University, Nada-ku, Kobe, Hyogo 657-8501, Japan; 4 RIKEN Center for Sustainable Resource Science, Tsurumi-ku, Yokohama, Kanagawa 230-0045, Japan

**Keywords:** genome editing, metabolic engineering, potato, steroidal saponins, TALEN

## Abstract

Potatoes produce steroidal glycoalkaloids (SGAs), toxic secondary metabolites associated with food poisoning. SGAs are synthesized by multiple biosynthetic enzymes. Knockdown of the *CYP88B1* gene, also known as *PGA3* or *GAME4*, is predicted to reduce toxic SGAs and accumulate steroidal saponins. These saponins not only serve as a source of steroidal drugs but are also anticipated to confer disease resistance to potatoes. In this study, we employed transcription activator-like effector nucleases (TALENs) for genome editing to disrupt *CYP88B1*. We introduced the TALEN expression vector via *Agrobacterium*-mediated transformation into seven potato lines. In six of these lines, disruption of the *CYP88B1* gene was confirmed. Liquid chromatography-mass spectrometry analysis revealed that SGAs were reduced to undetectable levels, corroborating the accumulation of steroidal saponins observed in previous knockdown studies. Our findings demonstrate the feasibility of generating low-toxicity potato lines through *CYP88B1* gene disruption using genome editing techniques.

## Introduction

Plants produce various specialized metabolites, many of which find applications in diverse fields such as medicine and cosmetics. However, some metabolites, like steroidal glycoalkaloids (SGAs) produced by potatoes (Friedman 2006) can be detrimental to human health. Potatoes rank as the fourth most widely cultivated crop globally. Nevertheless, improper cultivation, storage, and transportation can lead to the accumulation of toxic compounds, primarily SGAs, often resulting in foodborne illnesses characterized by symptoms such as nausea, vomiting, abdominal cramping, and diarrhea (Beals 2019). SGA synthesis is not restricted to the tuber, but occurs in all parts of the plant, including leaves, roots, and shoots (Friedman 2006).

**Figure 1:**
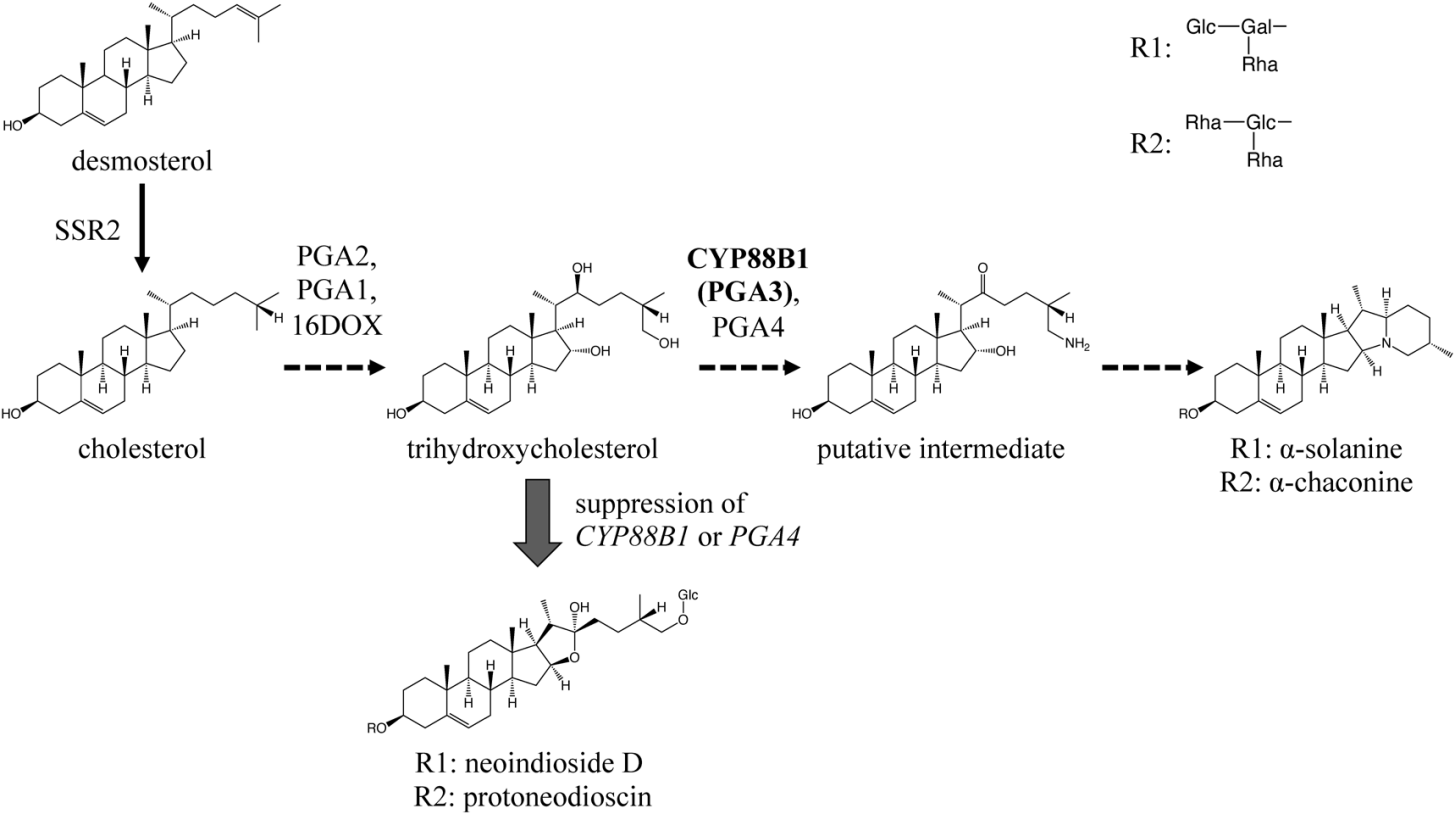
Figure 1. Biosynthetic pathway of steroidal glycoalkaloids in potato. Proposed biosynthetic pathway from desmosterol to α-solanine and α-chaconine. Solid and dashed arrows represent single and multiple enzymatic reactions in the pathway, respectively. The enzyme encoded by the *CYP88B1* (*PGA3*) gene that was targeted for genome editing in this study is shown in bold. The gray thick arrow indicates the accumulation of neoindioside D (25-*epi*-indioside D) and protoneodioscin by deactivation or suppression of *CYP88B1* or *PGA4*.

The SGA biosynthetic pathway in potatoes has been thoroughly analyzed, and many biosynthetic enzyme genes have been identified (Itkin et al. 2013; Nakayasu et al. 2017, 2021; Umemoto et al. 2016; Figure 1). Among these, sterol side chain reductase 2 (SSR2) plays a crucial role by catalyzing the branching reaction from the common plant metabolic pathway to SGA. Disrupting the SSR2 gene through genome editing has been shown to significantly reduce SGA content to approximately one-tenth of its original level (Sawai et al. 2014; Yasumoto et al. 2019). Moreover, the in planta suppression of gene expression through RNA interference (RNAi) targeting various biosynthetic genes has resulted in a considerable decrease in SGA content (Akiyama et al. 2023).

The cytochrome P450 monooxygenase gene *CYP88B1* (also known as *PGA3* and *GAME4*) has emerged as a potential candidate SGA biosynthetic gene through gene co-expression analysis, although its enzymatic activity remains uncharacterized (Itkin et al. 2013; Nakayasu et al. 2021). Knocking down *CYP88B1* via RNAi has been reported to significantly reduce SGAs such as α-solanine and α-chaconine while concomitantly accumulating steroidal saponins such as 25-*epi*-indioside D (also known as neoindioside D [Baur et al. 2022]) and protoneodioscin (Nakayasu et al. 2021). Although these steroidal saponins are considered less toxic to animals compared to SGAs, they can serve as valuable raw materials for steroids. Furthermore, their reported growth-inhibitory activity against the potato blight pathogen (*Phytophthora infestans*) (Baur et al. 2022) suggests that disrupting *CYP88B1* via genome editing could yield potato lines with reduced SGAs and enhanced accumulation of beneficial steroidal saponins, potentially conferring resistance to pests and diseases.

In this study, our objective was to employ *Agrobacterium*-mediated transformation to introduce an artificial restriction endonuclease transcription activator-like effector nuclease (TALEN) into potatoes, aiming to select genome-edited lines with disrupted *CYP88B1*.

## Materials and methods

### Generation of *CYP88B1* genome-edited potato lines

**Figure 2:**
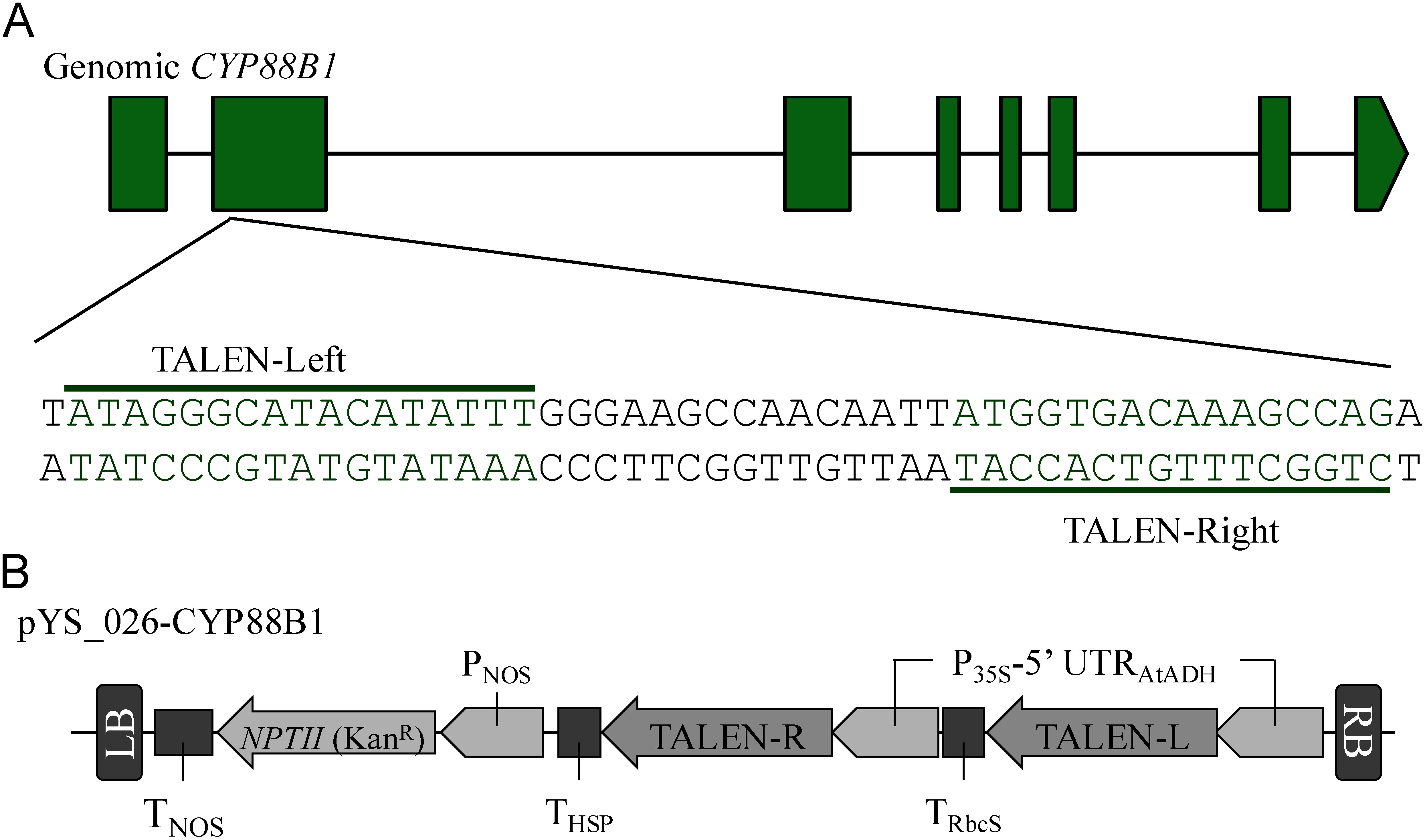
Figure 2. Schematic illustration of the T-DNA region of the platinum transcription activator-like effector nuclease (TALEN) expression construct. (A) Gene structure of potato *CYP88B1* and the target sequence of the designed TALEN. Green boxes and black line represent exons and introns of the *CYP88B1*. Target locations and sequences of TALENs are shown in the gene structure model. (B) Schematic illustration of the genome editing vector pYS_026-CYP88B1 used in this study. RB and LB, T-DNA right and left borders; P_35S_-5’UTR _AtADH_, Cauliflower mosaic virus 35S promoter 5’-untranslated region of *Arabidopsis thaliana alcohol dehydrogenase*; TALEN-L and TALEN-R, TALEN coding sequence for targeting *CYP88B1*. T_RbcS_, *Rubisco small subunit* terminator; T_HSP_, *heat shock protein* terminator from *A. thaliana*; P_NOS_, *nopaline synthase* promoter; *NPTII*, *neomycin phosphotransferase II*; T_NOS_, *nopaline synthase* terminator.

We designed a TALEN pair that targeted the *CYP88B1* sequence (Figure 2A). The expression vector for the Platinum TALEN, pYS_026-CYP88B1, was created according to a previous study (Figure 2B) (Yasumoto et al. 2019). The PtTALEN expression vector was introduced into *Agrobacterium tumefaciens* (EHA105 strain), and the resulting transformed *A. tumefaciens* cells and potato stem explants (cv. Sassy) were used to produce the transformed potatoes, as described in a previous study (Yasumoto et al. 2019). After rooting selection with kanamycin, the transformants were propagated on MS +3% sucrose medium, similar to the wild-type line. Genomic DNA was extracted from the resulting transformed and wild-type potatoes and used as a PCR template. The sequences surrounding the TALEN target sites were amplified using the primers pY419 (5′-GATCCAAATTCATTCCTTGATTTCTTTGC-3′) and pY420 (5′-ATCGACGAAAATATTTATCTTCTTCAATCGA-3′). The PCR products were analyzed using a microtip electrophoresis machine (MultiNA, Shimadzu Co., Japan). The PCR products were cloned into the pJET1.2/blunt vector (Thermo Fisher Scientific, USA), and DNA sequence analysis was performed to confirm the target sequence.

### Metabolite analysis of *CYP88B1* genome-edited lines

The amounts of SGAs and steroidal saponins in the seedlings were quantified using liquid chromatography-mass spectrometry (LC-MS), as previously reported (Nakayasu et al. 2021). In summary, approximately 4-week-old shoots and leaves of the sterile seedlings were lyophilized, ground, and about 2 mg was placed into a vial. The sample was then extracted three times with 200 µl of methanol and analyzed via LC-MS.

## Results

### Targeted genome editing of transgenic potatoes

**Figure 3:**
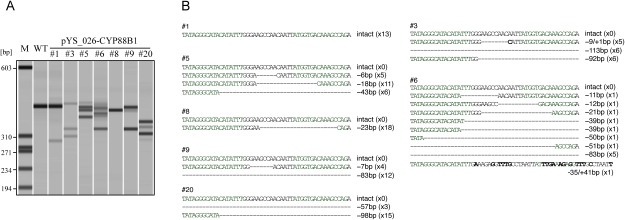
Figure 3. Validation of targeted mutagenesis in transgenic potato lines. (A) Amplification of the sequences surrounding the TALEN target sites in *CYP88B1* by polymerase chain reaction (PCR). Target sequences were amplified from genomic DNA extracted from either wild-type (WT) or pYS_026-CYP88B1, the TALEN expression vector, transformed potato lines (#1, #3, #5, #6, #8, #9, and #20). M, DNA marker (PhiX174 DNA-HaeIII, New England Biolabs, USA). (B) Target *CYP88B1* nucleotide sequence in transgenic potato lines. Multiple alignments of *CYP88B1* target sequence and PCR products were amplified from transgenic potato lines. TALEN-recognized sites are highlighted in green. Dashes and bold letters in the sequence alignment indicate deletions and insertions, respectively. The right side of the nucleotide sequence shows the pattern of mutations (insertion-deletion) and the number of detected sequences.

We generated seven independent transgenic potato plants harboring a TALEN expression vector targeting *CYP88B1* gene. Genomic DNA extracted from these transformed lines was used as a template to amplify the target sequence of TALEN by PCR, and analysis using a microchip electrophoresis system revealed a band pattern in all lines that was different from that of the wild-type (Figure 3A). In all samples except sample #8, multiple bands were detected, suggesting the formation of heteroduplexes at the target site, which were not observed in the wild-type sample. In transgenic line #8, only one band was observed, similar to that of the wild type; however, it was shorter than the wild type, suggesting a targeted deletion at the target site. After the PCR analyses, the target nucleotide sequences were confirmed by Sanger sequencing. In transgenic line #1, only wild-type sequences were detected, and no mutagenesis was identified, whereas in the other six transformed lines, no wild-type sequences were found, and a wide variety of deleted sequences ranging from 7 bp to approximately 100 bp were detected (Figure 3B).

### Metabolite changes in *CYP88B1* genome-edited potatoes

**Figure 4:**
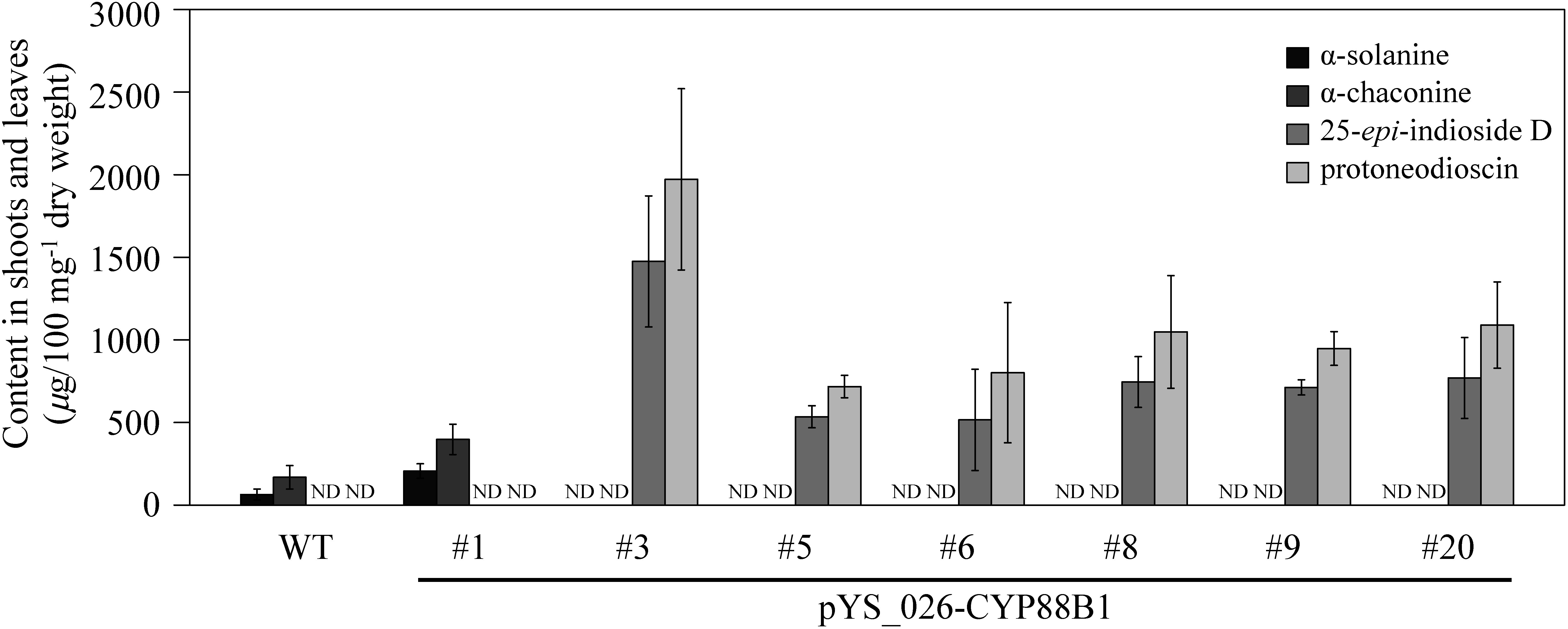
Figure 4. Steroidal glycoalkaloids and saponins in *CYP88B1* genome-edited potatoes. The α-solanine, α-chaconine, 25-*epi*-indioside D, and protoneodioscin levels in transgenic potato lines. The metabolite levels in leaves and stems from in vitro aseptic cultures of either wild-type (WT) or pYS_026-CYP88B1, TALEN expression vector, transformed potato lines (#1, #3, #5, #6, #8, #9, and #20) were analyzed using LC-MS. Error bars indicate the standard deviation of three replicates. ND means not detected.

Metabolites were extracted from all transformants and wild-type potatoes and quantitatively analyzed for SGA (α-solanine and α-chaconine) and steroidal saponins (25- *epi*-indioside D, and protoneodioscin) via LC-MS (Figure 4). In transformant line #1, wherein the mutated *CYP88B1* was not detected by Sanger sequencing, the same level of SGAs as in the wild type accumulated. In other transgenic lines where intact *CYP88B1* was not detected, SGAs were not detected, and steroidal saponins were detected to a greater extent than in wild-type SGA.

## Discussion

SGAs are significant secondary metabolites in potatoes, and there is a pressing demand for developing potato lines with reduced SGA content in breeding programs. Previous studies attempted to achieve this by knocking out *SSR2*, the biosynthetic enzyme gene responsible for SGA production, using genome editing. However, due to the presence of *SSR1*, a paralog of *SSR2*, the resulting mutants exhibited a phenotype with reduced, but not eliminated, SGA levels (Sawai et al. 2014). In this study, we targeted another SGA biosynthetic enzyme gene, *CYP88B1*, for genome editing using TALEN. All seven lines transformed with the TALEN expression vector via *Agrobacterium*-mediated transformation showed evidence of targeted mutagenesis in PCR tests (Figure 3). Subsequent Sanger sequencing confirmed changes in target sequences across six lines, indicating disruption of gene function. Line #1 displayed a mutation in PCR tests, although sequencing analysis did not confirm it, likely due to a low mutation rate in this line. Metabolite analysis revealed the absence of SGAs in the six lines with confirmed mutations, indicating successful disruption of the SGA biosynthetic enzyme function (Figure 4). Instead, these genome-edited lines exhibited an accumulation of steroidal saponins, consistent with findings in RNAi lines (Itkin et al. 2013). Utilizing transient expression systems with *Agrobacterium*, we generated genome-edited potatoes devoid of foreign genes (Umemoto et al. 2023; Yasumoto et al. 2020). Genome-edited lines that do not contain foreign genes are permitted to be grown in fields without being subject to Cartagena law regulations, if the appropriate documentation is submitted to the regulatory authorities (Nishihara and Muranaka 2023). While the *CYP88B1* genome-edited lines reported here represent stable transformants, the transient expression system holds promise for the creation of new genome-edited lines that can be grown in the field and are completely free of foreign nucleic acids, devoid of toxic SGAs, or enriched with beneficial steroidal saponins. These lines could serve as novel resources for steroid production and valuable breeding materials imparting resistance to potato blight disease.

## Abbreviations

LC-MS: liquid chromatography-mass spectrometry
PCR: polymerase chain reaction
RNAi: RNA interference
SGAs: steroidal glycoalkaloids
TALEN: transcription activator-like effector nuclease
